# HPLC fractionation with immunoassay of steroids from nipple aspirate fluid

**DOI:** 10.1016/j.mex.2022.101775

**Published:** 2022-06-26

**Authors:** Richard E. Heinz, Robert T. Chatterton

**Affiliations:** a Department of Surgery, Northwestern University Feinberg School of Medicine, Chicago, IL 60646; b Department of Obstetrics/Gynecology and the RH Lurie Comprehensive Cancer Center, Northwestern University Feinberg School of Medicine, Chicago, IL 60646

**Keywords:** Liquid biopsy, Breast cyst fluid, Adipose tissue, Steroid quantification, Low volume sample, High-performance liquid chromatography

## Abstract

Fractionation of steroids allows for multiple assays to be run on a single low volume liquid biopsy, whereas performing the same number of assays without fractionation would require increasing the sample volume by dilution, rendering the concentration of steroids below the level of detection for most, if not all, downstream assays. Briefly, steroids are extracted from a biofluid sample using solvent phase extraction to separate the aqueous (conjugated) steroids from the non-aqueous (non-conjugated) steroids in the organic phase. The latter is further separated by high-performance liquid chromatography (HPLC) and collected in an automated fraction collector based on the UV detection of internal standards. Commercially available immunoassays are then used to quantify the < ng/ml concentrations of steroids in each fraction. This protocol was designed for small samples of nipple aspirate fluid (minimum 2 µL), but it can be modified to fractionate steroids from homogenized solid tissue samples or other liquid biopsies. Included in this protocol are precautions to help ensure reproducibility and minimize matrix effects and other errors of measurement, given that samples requiring fractionation are fundamentally precious and, like other quantitative procedures of small samples, can be prone to contamination by solvent residues and other factors.

• The method permits quantitative analysis of multiple steroids from very small volumes of biofluid.
• Fractionation by HPLC provides a highly purified sample for quantification.
• The immunoassay end point provides specificity without expensive equipment.

## Graphical abstract

**Figure:**
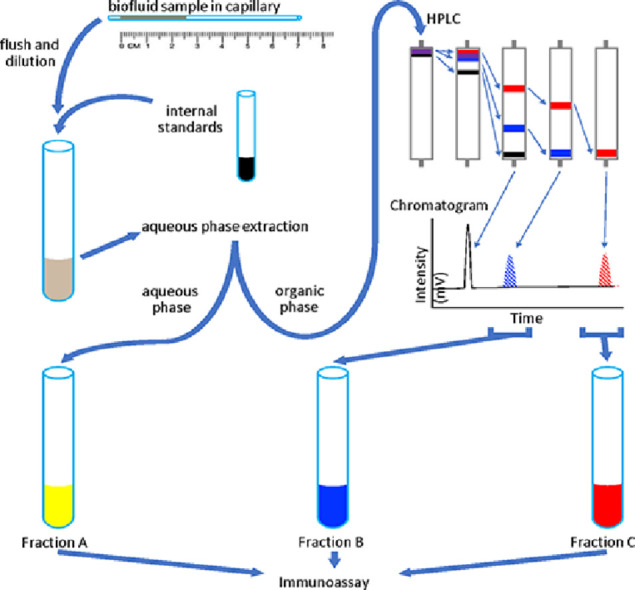

**Specifications table**

**Table:** 

Subject Area;	Biochemistry, Genetics and Molecular Biology
More specific subject area;	*Analysis of steroid hormones by HPLC and immunoassay*
Method name;	*Method for analysis of steroids in nipple aspirate fluid*
Name and reference of original method;	N/A
Resource availability;	HPLC (Hitachi) with degasser, column heater, and in-line UV detector Automated fraction collector Nitrogen evaporator Speed vacuum concentrator Absorbance plate reader Scintillation counter

## Introduction

This procedure was designed to assay steroid hormones in the very small volumes of nipple aspirate fluid (NAF) obtained from women who are at high risk for breast cancer. The initial study using this specific protocol was published in 2015 [1] with additional studies thereafter [2], [3], [4]. The steroids being measured are present in concentrations of less than ng/ml, thus requiring a more sensitive method of quantification than UV absorbance. We chose to use immunoassays for this purpose. With immunoassays as the endpoint, one must be concerned with contamination and matrix effects which result in interference with binding of the analyte to the antibody. Such interference could result in calculated values that are greater than the actual amounts of analyte in the sample. The sample itself may have matrix substances that compete for the antibody, and contamination from solvents must be scrupulously avoided. Purification of the sample by high performance liquid chromatography (HPLC) is an excellent procedure for minimizing effects of interfering substances.

The alternative to immunoassays is mass spectrometry. By contrast with immunoassays, contamination and matrix effects can cause suppression of the signal measured by mass spectrometry, leading to values that are less than the actual amount of analyte [5].

In the present HPLC-immunoassay method we evaluate all known sources of variation, and where interfering substances cannot be eliminated by sample purification, they are accounted for in the calculated final values.

## Method Details

### Sample Acquisition and Storage

All sample collection was conducted by methods approved by the Institutional Review Board of Northwestern University, and all subjects consented to the procedure. Nipple aspirate fluid (NAF), collected in nonheparinized glass capillary tubes and sealed with clay plugs, is brought to the laboratory on ice. Upon receiving the NAF specimens in the laboratory, cut off the clay plug furthest away from sample to release any pressure, and briefly centrifuge to remove any air bubbles (seen and unseen). Use a ruler to measure the cumulative length of liquid in mm from the edge of the remaining clay plug to the interface between the sample and the air as illustrated in the top left of the graphical abstract. With standard capillary tubes, each mm equals one µL. This measurement will dictate how samples are processed, but final concentration of steroids may be expressed by volume or in relationship to total protein. Carefully cut off the remaining clay plug and flush out the NAF into a cryovial using 200 µL of Dulbecco's phosphate buffered saline (DPBS). DPBS is used to retain physiological pH for potential enzymatic assays and to better rinse out the capillary tube. Triturate the diluted NAF through the capillary tube until completely rinsed out. If necessary, NAF can also be removed by using a plunger from a 25 µL Hamilton syringe. Store diluted NAF in a sealed vial at -80°C to preserve the sample until ready to process. The overall procedure is presented in the graphical abstract.

### Prepare an Internal Standard Cocktail

An in-line UV detector is not sensitive enough to measure the ng/mL range of steroids in NAF being fractionated by HPLC, but it can be used to detect exogenously added internal standards to determine percent recovery of the non-aqueous (unconjugated) steroids. It is important to determine percent recovery because it is not possible to inject the full volume of sample into the HPLC. A dead volume is necessary to ensure no air gets injected. Samples are prepared to a volume of 100 µL and 75 µL is injected. Therefore, we expect a recovery of about 75 percent, but this varies depending on how much evaporation of the sample takes place prior to injection and how accurate the injection volume is.

For an internal standard, we use a stock cocktail of 25 µg/mL of dexamethasone (DEX) and 25 µg/mL of prednisone acetate (PRED ACE) in 30% methanol prepared with HPLC grade water. Both of these are synthetic steroids with no cross-affinity for the anti-steroid antibodies used to assay the endogenous steroids, and therefore will not impact their quantification even if elution times overlap. They are also equally soluble in the extraction medium as the compounds of interest. PRED ACE serves as the primary internal standard used to determine percent recovery for a sample while DEX serves as a backup to PRED ACE. The graphical abstract presents the internal standard cocktail as one standard, colored in black. The illustration shows how the internal standards are added to the diluted NAF, are extracted along with the other non-aqueous steroids, and how they are measured by the in-line UV detector.

A plot of area vs. mass of the internal standard verifies linearity and establishes a formulaic relationship. For each batch of samples, prepare a new calibration curve of each internal standard by running 5 serial dilutions of cocktail from 10 µg/mL to 0.625 µg/mL in duplicate to establish average area under both peaks of the resulting chromatogram. Dilutions are made with wash solution (water, methanol, and acetonitrile at a ratio of 3:1:1). Injection of 75 µL contains between 750 ng to 46.88 ng of each standard, which is detectable by the UV detector. Our UV detector calculates area under the peak, so we can plot area vs mass. After we inject samples, the resulting area can be interpolated to calculate a percent recovery, which factors into our final calculations of concentration of steroid in NAF.

### Prepare an external reference control

Prepare a large pool of samples that contain immunoassay detectable levels of the steroids of interest to use as an external reference control. This control should yield reproducible results and can be used as an additional way to determine recovery of each sample as well as a way to track and adjust for inter-batch variability. Ideally, the external reference control should be of similar composition to the samples being processed because recovery won't be identical across different matrices. For NAF, we use a pool of spiked breast cyst fluid (BCF) that is aliquoted and stored at -80°C. If any analytes of interest are not present at detectable concentrations in the BCF, we spike the pool with exogenous steroid to bring the concentration to a detectable level when processed the in a volume of 10 µL. Preparation of an external reference control is not represented in the graphical abstract, but once prepared, it follows the same procedural steps as NAF, starting from the aqueous dilution step illustrated on the top left of the graphical abstract, and which is further described in the following section of the method.

### Prepare aqueous dilutions

Prepare aqueous (Aq) dilutions of each sample according to the volume of NAF obtained. When available, 10 µL is considered an ideal volume of NAF where most analytes will be detectable after fractionation, but smaller volumes, as low as 2 µL may be processed for at least some steroids. Dilution with buffer is necessary to have sufficient volume for all the desired assays to be run on the aqueous fraction.

• **10 µL ≤ NAF ≤ 20 µL**: Briefly centrifuge the thawed vial of diluted NAF, vortex lightly, and remove the entire sample to a 13 × 100 mm glass culture tube labeled with “Aq” and a sample identification number (ID). Rinse out the vial with enough phosphate buffered saline (PBS) to bring the diluted NAF samples to a final volume of 0.5 mL based on the volume measured in the capillary and the additional 200 µL of DPBS used to flush it out of the capillary. Add rinse to the culture tube with the sample.

Example: If a sample originally had 14 µL of NAF it would have been initially diluted to 214 µL. Use all of the diluted NAF. Add 286 µL of PBS to bring the final volume to 0.5 mL.

• **NAF < 10 µL**: For small volumes, samples are at risk of being too dilute to quantify for some analytes if processed the same as larger volumes. To help ensure detectable concentrations, reduce all fraction volumes by half and only reconstitute samples in enough buffer to assay in singlicate. Briefly centrifuge the thawed vial of diluted NAF, vortex lightly, and remove the entire sample to a 13 × 100 mm glass culture tube labeled with “Aq” and the sample ID. Rinse out the vial with enough PBS to bring the diluted NAF samples to a final volume of 0.25 mL based on the volume measured in the capillary and the additional 200 µL of DPBS used to flush it out of the capillary. Add rinse to the culture tube with the sample. Label the tube with an “H” (for half) as a reminder that it needs to be assayed in singlicate.

Example: If a sample originally had 4 µL of NAF, it would have been initially diluted to 204 µL. Use all of the diluted NAF. Add 46 µL of PBS to bring the final volume to 0.25 mL.

• **NAF > 20 µL**: Briefly centrifuge the thawed vial of diluted NAF, vortex lightly, and transfer half of the sample to a 13 × 100 mm glass culture tube labeled the same way as with the 10 µL 20 µL NAF samples. The correct volume to retain for extraction is determined by taking the sum of the volume measured in the capillary and 200 µL (volume of DPBS added) then dividing the result by 2. Continue to store the remaining half at -80°C for backup. Bring the retained portion of diluted NAF to a final volume of 0.5 mL.

Example: If a sample originally had 44 µL of NAF, it would have been initially diluted to 244 µL. Use only half of the diluted NAF, which is 122 µL. Add 378 µL of PBS to bring the final volume to 0.5 mL.

In addition to samples, we also prepare aqueous dilutions of positive and negative controls. A method blank (BLK) will serve as the negative control. The BLK is treated like a NAF sample. It is prepared by adding 50 µL PBS to 200 µL of DPBS and extracted the same as NAF samples. All Aq fraction tubes containing BLKs will be processed like a low volume sample and expected to have values below quantification. Breast cyst fluid (BCF), as mentioned earlier, will be used as an external reference (positive) control. The BCF sample is treated like a NAF sample. It is prepared by adding 290 µL PBS to 200 µL DPBS and spiked with 10 µL BCF. The positive and negative controls are used in the final calculations of steroid concentrations in NAF, and we noticed percent recovery changes enough after running about 4 samples that it is best to include at least one set of controls for every 4 samples. Because these controls are essential to our final calculations, we always prepare at least two sets in case one is needed as a backup. We use the same labeling notation as with the samples, substituting BLK or BCF for the sample ID. To designate one set of controls by another within the same batch, we amend a number after “BLK” or “BCF”. For example, BCF1, BLK1, BCF2, BLK2, etc.

### Aqueous phase extraction

Add 10 µL of prepared internal standard cocktail (DEX and PRED ACE) to all diluted samples (including controls). This ends up being an addition of 250 ng of each internal standard. The graphical abstract illustrates this step as the tube of black internal standard going into the brown colored diluted sample. The aqueous (conjugated) steroids are separated from the non-aqueous (non-conjugated) steroids by solvent extraction. This is done by adding 1 mL of HPLC grade ethyl acetate:hexane (3:2) that has been equilibrated with HPLC grade water to the diluted sample. Equilibration of the acetate:hexane with water maintains the volumes of each solvent. Vortex the sample slowly, then centrifuge for 5 minutes at approximately 2500 RCF to separate the aqueous phase from the organic phase, as illustrated in the center of the graphical abstract. Transfer the top (organic) layer to 12 × 75 mm polypropylene tubes, which give better recovery of steroids than glass tubes, and label with the samples. Repeat this step twice for a total of 3 mL of extract, being careful not to include any aqueous (Aq) phase with the organic solvent. It is important to wear gloves and use filter tips since risk of contamination with non-polar hormones will increase when working with non-polar solvents. Evaporate any residual ethyl acetate: hexane from the aqueous fractions by placing them in a 50°C water bath under nitrogen (N_2_). Samples are done when the ethyl acetate:hexane is no longer detectable in the aqueous fraction.

Use of the N_2_-evaporator poses a potential risk for contamination. It is important to replace the inline filter at least every 6 months, wipe the needles down with methanol twice before inserting them into the sample tube, avoiding any contact between the needle and the sample, and gently turn on the gas to avoid splashing the sample.

To help prevent degradation of the aqueous sample, add 2 µL of protease inhibitor cocktail (Sigma P8340) (diluted 1:2 in DMSO) to the 0.5 mL Aq fractions and 1 µL to the 0.25 mL Aq fractions. Centrifuge the Aq fractions briefly to pool any moisture that may have condensed on the side walls while in the evaporator. Since some of the water in the Aq fraction may have evaporated and left the tube, measure the volume with a manual pipette, and add HPLC grade water as necessary to bring the Aq fraction back to the recorded volume of 0.5 mL or 0.25 mL. The Aq fractions can then be capped and stored at -20°C for no more than 1 month before assaying. The Aq fraction is represented in the graphical abstract as Fraction A in yellow.

We assay our Aq fraction for dehydroepiandrosterone sulfate (DHEA-S), total protein, and insulin-like growth factor-1 (IGF-1), but this fraction could also be used for assaying other water-soluble substances. The lower limit of detection of these analytes by assays available to us is 62.5 ng/ml for DHEA-S (Beckman-Coulter, DSL-2700), 0.156 ng/mL for IGF-1, and 0.781 µg/mL for protein. The DHEA-S and IGF-1 assays used are radio-immunoassays and the protein assay is a fluorescence- based assay.

The next two steps called HPLC Preparation and Automatic Fraction Collector Calibration are not indicated in the graphical abstract but contain important actions to ensure reproducibility of our method.

### HPLC preparation

For our analytes of interest, the Supelco Cat #53827-U Ascentis Express C18 column 10 × 4.6 cm 2.7 µm particle size provides the best resolution, but exploring other options is encouraged depending on the analytes being separated. A guard column made of the same material is also recommended, so for the Supelco Cat #53827-U column, we use the Supelco Cat #53508 guard column. Replace the guard column when the chromatogram of the internal standard peak starts to trail or form a shoulder. Replace the column when the system experiences abnormally high pressure that isn't resolved by purging the system. It is imperative that the HPLC equipment receive professional servicing at recommended intervals to ensure the performance stays consistent and to replace all consumable parts before they fail.

The buffers and the gradient used can also be modified depending on what analytes are being separated. For our purposes, three buffers are prepared. Buffer A is 60% 15 mM phosphate buffer, pH 6, 20% methanol, and 20% acetonitrile. Buffer B is 50% acetonitrile and 50% methanol. Wash solution is the same as buffer A, but without the salt. Buffers are made with HPLC grade reagents and degassed with an in-line degasser in order to remove dissolved gas from the buffer which would otherwise effect elution time consistency. We made 4 L volumes of buffer A at a time, stored at 4°C, to maintain better consistency between batches.

To prepare the samples, completely evaporate ethyl acetate:hexane from the organic phase of NAF extractions by placing the polypropylene tubes in a 50°C water bath under N_2_, using the same precautions mentioned earlier regarding the N_2_-evaporator. This takes about 10 to 15 minutes. Re-dissolve the sample residues in 40 µL buffer B and vortex briefly. Add 60 µL HPLC grade water for a final volume of 100 µL. The purpose of adding Buffer B and water separately is so that Buffer B can completely solubilize the steroids, and the water can bring the final volume to 100 µL and bring the final concentration of methanol in the sample to the same as the concentration that is being added to the column immediately before and after the sample. Because it would not be possible for the autosampler to draw up every last drop without also injecting air into the system, program the autosampler to draw up 75 µL for HPLC injection. Transfer the sample to a glass polyspring insert labeled with the sample ID. Without the polyspring insert, we wouldn't be able to inject the entire volume of sample because of the required dead volume. Place the inserts into amber HPLC vials and seal with a cap and silicone septum to help prevent evaporation of sample. We found higher background on some analytes when we tried rubber septums, so we stick with silicone. Samples can be kept at 4°C until ready to be fractionated, for up to a few days.

Initialize the HPLC system as per manufacturer instructions. Because temperature changes can affect elution times, use a column heater to keep the column a consistent temperature. We opted for 26.2°C which is deliberately about 5 degrees above room temperature to provide a buffer against any subtle raise in temperature over the course of several hours. Immediately before use, purge all components of the HPLC prior to the column with buffer B until all bubbles are drained. Then purge the autosampler, followed by the column itself. Next, prime the system with buffer A until the chromatogram baselines, at which point the UV detector will need to be re-zeroed.

We developed a program to best separate our steroids of interest using the C18 column. This program uses 100% buffer A for the first 40 minutes to elute the steroids in order of more polar to less polar, a linear gradient to 50% buffer B from 40-50 minutes to gradually change buffer concentration, 50% buffer B from 50-55 minutes to elute the most non-polar steroids, a linear gradient to 100% buffer B from 55-60 minutes to purge the column of any residual residues, a reverse linear gradient back to 100% buffer A from 60-65 minutes to gradually change buffer concentration back to the original, and lastly, 100% buffer A from 65-75 minutes to clean and re-prime the system between samples. The flow rate should be set to 0.6 mL/min. Make sure to empty the waste container, and that it is large enough to handle the length of time that the batch will be fractionated.

### Automated fraction collector calibration

The automated fraction collector can collect effluent at specific times during the HPLC program. Before each batch of samples, determine the retention times of four calibration standards in order to calibrate the automated fraction collector. This is necessary because elution times may vary slightly from one batch of HPLC buffer to the next, and it serves as a quality control prior to set up of the fraction collector. The calibration sample is a cocktail of 4 synthetic steroids that elute at selected times in the range of elution of the endogenous steroids of interest. The cocktail includes PRED ACE (which was also used as an internal standard), norethindrone (NOR), dexamethasone acetate (DEX ACE), and norethindrone acetate (NOR ACE). These 4 synthetic steroids are each at a concentration of 2.5 µg/mL in wash solution (water, methanol and acetonitrile at a ratio of 3:1:1). This is a concentration that is high enough to visualize by UV absorbance with the in-line UV spectrometer. PRED ACE will be the first peak around 25-30 minutes, followed by NOR at 29-34 minutes, DEX ACE at 46-50 minutes, and NOR ACE at 53-58 minutes. Only 2.5 µg/mL is needed instead of the 25 µg/mL used as an internal standard because the calibration standard cocktail is just to determine elution times, not percent recovery.

**Fig. 1:**
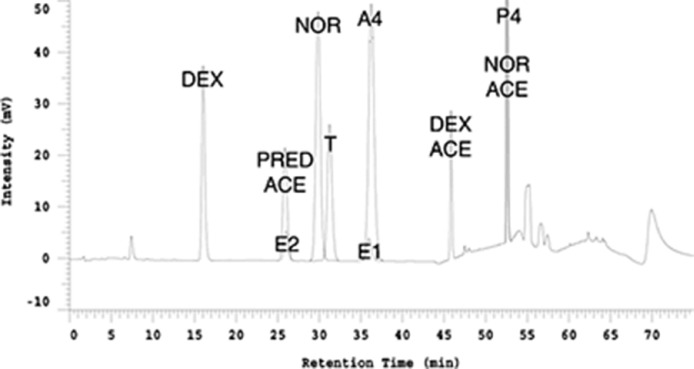
Chromatograph of analytes of interest in relation to calibration and internal standards. Superimposed chromatographs of estrogens (read at 280 nm) and everything else (read at 240 nm). Shown are dexamethasone (DEX), prednisone acetate (PRED ACE), estradiol (E2), norethindrone (NOR), testosterone (T), androstenedione (A), estrone (E1), dexamethasone acetate (DEX ACE), progesterone (P4), and norethindrone acetate (NOR ACE). A standard curve will be created for the 2 internal standards DEX and PRED ACE prior to each batch, and those two peaks will appear in the chromatograph for every sample. Peaks for the 4 synthetic steroids, DEX, PRED ACE, NOR and NOR ACE will only appear in the fraction collector calibration. NOR and NOR ACE are only included in the fraction collector calibration to extend the range and are not added to the samples as internal controls.

We use the elution times of each calibration standard, and their relationship to the steroids of interest, to set the collection times on the fraction collector. The relationship of the steroids to each calibration standard is shown in Fig. 1, and this chromatograph only needs to be determined once. Estrogens can be read at 280 nm, and the other steroids are read at 240 nm. Fig. 1 was created by overlaying chromatographs from both wavelengths. When actual samples are run on the HPLC, only the two internal standards of (DEX) and (PRED ACE) will be visible on the chromatograph, which is why this figure had to be created to know where everything else elutes. One analyte of interest, dehydroepiandrosterone (DHEA), does not absorb UV light in the range of the other steroids, so elution time was detected by sulfuric acid charring of 0.5 min fractions from the range of 35-50 minutes spotted on a thin layer chromatography plate. Again, this only had to be done once, and it showed that DHEA elutes between 40 and 42 minutes. For all the non-aqueous (unconjugated) steroids, the following general rules were derived as shown in Fig. 1 to determine the collection time windows that are re-calibrated for each batch:

• Testosterone (T) elutes immediately after NOR. The collection window for T should start at the left edge of the NOR peak and end 5 minutes later.
• The Estradiol (E2) collection window should end 0.5 min before the start of T and begin 5 min earlier. E2 elutes at the same time as the internal standard prednisone acetate, so when the sample is being collected the prednisone acetate peak should be visible at the same time the E2 collection occurs.
• Estrone (E1) and Androstenedione (A4) elute together. The collection window for E1/ A4 should be immediately after T and last for 5 min (there is no cross-reactivity of these steroids in the immunoassay)
• The collection window for DHEA is in a 5-minute period between the E1/A4 window and DEX ACE. If the space between the two is less than 5 minutes, just start the collection window immediately after the E1/A4 collection.
• Progesterone (P4) elutes at the same time as NOR ACE. Set up the collection time to start 2.5 min. before the center of the NOR ACE peak and end 2.5 min. later for a total time of 5 min.

Once a calibration set is run for a new batch, set the fraction collector to collect at the times derived from the calibration. We collect 5 fractions per sample and for 5 minutes per fraction. Pre-label 13 × 100 mm glass tubes that the fractions will be collected into with the name of the analyte, the sample ID and “H” for any BLK samples or samples derived from less than 10 µL of NAF. Arrange the prelabeled tubes in the collection rack of the fraction collector so that they follow the order that the fraction collector will disperse.

### HPLC fractionation

It is good to position one of the BCF controls first in the batch, in case there are any errors in the set up that can be caught early. When starting the first sample of a batch, check that the septum of the HPLC vial was pierced by the autosampler, that no more than 25 µL remains in the polyspring insert after being injected into the system, that the pressure isn't too high or low, that the collection tubes were arranged in the correct order, that the fractions are being dispensed correctly, that the fractionation volumes are as expected, that the live readout of the buffer ratio being used at any given moment matches what was programed, that the two internal standards elute when expected, and the next sample gets injected immediately after the first sample is complete. Between batches, run 100% buffer B for an extended amount of time to keep the column clean and prevent salt in buffer A from depositing. Whenever the column is not in use, there should only be buffer B in the system. There are several physical limits to how many samples can be run in one batch, but our system works well processing 16 samples overnight, including 3 sets of controls and 10 samples of NAF.

The graphical abstract shows how a combined sample of 2 steroids (blue and red), along with an internal standard (black), separate from each other as they pass through the HPLC column. They are separated cleanly by the automated fraction collector. While the internal standard appears as a black peak in the chromatogram, we've only used a partially colored peak to show the location of the 2 steroids, which are not visible on the chromatogram, but which are eluted in fractions B and C. (The actual separation of the endogenous steroids involves collection of all 5 fractions from the HPLC).

### Fraction processing

Evaporate fractions as soon as possible following fractionation. It is ok to store them sealed with parafilm for a day or two at 4°C. We used a speed vacuum concentrator to evaporate the P4, E2, and T fractions, and the N_2_-Evaporator for the E1/A4 and DHEA fractions. After evaporating the HPLC buffer from each fraction, reconstitute them in enough volume of steroid buffer or bovine serum albumin (BSA) buffer to assay them in duplicate (or singlicate where NAF volume was less than 10 µL). Steroid Buffer is 0.1% gelatin in 0.4% sodium phosphate monobasic, 0.9% Sodium Chloride (NaCl), and 1.1% sodium phosphate dibasic, pH 6.95 with 0.07% Kathon added as a preservative. BSA Buffer is 0.1% bovine serum albumin in PBS, pH 7.4 with 0.07% Kathon added as a preservative. With the exception of the E2 fraction, we found that we could reconstitute each fraction in enough buffer to perform the assay twice if needed. Cap each fraction and shake at room temperature for 1 hour to fully resuspend the steroids in solution. Store fractions at -20°C along with the Aq fractions for no more than 1 month before performing assays. Store samples at -80°C if assays will not be performed within 1 month.

The assays we use to measure the amount of steroid in each fraction are either radio-immunoassay or enzyme immunoassays. The lower limit of detection of these steroids by the methods used by us is 6.25 pg/ml for E2 (Beckman Coulter DSL-2700), 6.25 pg/ml for E1 (Beckman Coulter DSL-9700), 150 pg/ml for P4 (Salimetrics 1-502-5), 19.53 pg/ml for A4 (Beckman Coulter DSSL-3800), 75 pg/ml for T (Salimetrics 1-2402), and 0.23 ng/ml for DHEA (Alpco 20-DHEHU-E01). The E2 assay used is a radio-immunoassay, and the rest are enzyme immunoassays.

### Calculating concentration of steroids in NAF

The HPLC should be programed to read UV absorbance at 240 nm and print out a chromatogram for each sample, showing peaks for the two internal standards DEX and PRED ACE. Use the area under the PRED ACE peak to determine percent recovery based on the standard curve of PRED ACE which has been plotted prior to each batch. If the PRED ACE peak is not symmetrical, use the DEX peak and its standard curve instead. The blank value is subtracted from the sample value, and the recovery is calculated from the internal standard. In a recent study the values of the BLK fractions were: E2, 2.72 ± 2.49 SD pg/ml; E1, 4.00 ± 4.63 SD pg/ml; P4, 8.15 ± 3.27 SD ng/ml; A4, 5.27 ± 4.36 SD ng/ml; T, 3.43 ± 3.35 SD ng/ml; DHEA, 0.84 ± 1.27 SD ng/ml; and DHEAS from the aqueous fraction was 0.22 ± 0.88 µg/ml.

An additional batch correction is made using the external reference controls (BCF samples) that were run with each batch. The concentrations in the external reference control should be consistent between batches, so a further normalization should be performed on all samples within a batch by adjusting the sample values for differences in the mean value of BCF standards from the accepted norm.

## Declaration of Competing Interest

The authors declare that they have no known competing financial interests or personal relationships that could have appeared to influence the work reported in this paper.
